# Incidental Detection of a Well-Differentiated Neuroendocrine Tumor of the Gallbladder: A Case Report

**DOI:** 10.7759/cureus.101754

**Published:** 2026-01-17

**Authors:** Riku Yamamoto, Shunsuke Sakuraba, Kosaku Nihei, Kenichiro Tanaka, Tomoaki Ito

**Affiliations:** 1 Department of Surgery, Juntendo University Shizuoka Hospital, Shizuoka, JPN

**Keywords:** case report, cholecystectomy, gallbladder, neuroendocrine neoplasm (nen), neuroendocrine tumor (net) g1

## Abstract

Neuroendocrine tumors (NETs) of the gallbladder are uncommon and are often difficult to diagnose preoperatively due to their nonspecific clinical and imaging findings.

A 39-year-old man presented with right upper quadrant abdominal pain without fever, jaundice, or signs of peritoneal irritation. Abdominal ultrasonography revealed a 9 mm polypoid lesion in the neck of the gallbladder, and contrast-enhanced computed tomography demonstrated an 8 mm slightly enhancing lesion with gallbladder wall thickening consistent with cholecystitis. Blood tests showed no elevation of inflammatory markers. Although initial observation was considered, the patient experienced recurrent biliary colic-like pain. We suspected that the polyp located in the gallbladder neck was causing intermittent obstruction of the cystic duct, leading to these symptoms. Laparoscopic cholecystectomy was performed under a preoperative diagnosis of a benign gallbladder polyp. Histopathological examination showed uniform proliferation of tumor cells confined to the mucosa with occasional glandular structures in the background of chronic cholecystitis. Immunohistochemical analysis revealed positivity for neuroendocrine markers (chromogranin A, synaptophysin, and CD56) with a Ki-67 labeling index of <1%. Based on these findings, the tumor was diagnosed as a well-differentiated NET of the gallbladder.

We experienced a case of well-differentiated NET incidentally diagnosed after laparoscopic cholecystectomy for a gallbladder polyp. Since cases of early-stage gallbladder NETs are rare, their clinical behavior and long-term prognosis remain poorly understood. This case underscores the need for continued case accumulation and careful postoperative follow-up to establish appropriate management strategies for this rare entity.

## Introduction

Neuroendocrine neoplasms (NENs) comprise a heterogeneous group of tumors, including well-differentiated neuroendocrine tumors (NETs) and poorly differentiated neuroendocrine carcinomas (NECs). They most commonly arise in the gastrointestinal tract, followed by the lungs and pancreas [1], with an estimated annual incidence of approximately 5-6 cases per 100,000 population [2]. In contrast, gallbladder NENs account for approximately 0.5% of all NENs [3]. This low incidence is thought to be related to the limited distribution of native neuroendocrine cells in the normal gallbladder mucosa [2,3]. Most reported gallbladder NENs are NECs or mixed adenoneuroendocrine neoplasms, which show aggressive clinical behavior and are often diagnosed at an advanced stage. These tumors differ substantially from conventional gallbladder adenocarcinomas in terms of histogenesis, biological behavior, and prognosis.

Here, we report a case of a well-differentiated gallbladder NET classified as NET G1 [4]. The tumor was incidentally diagnosed after laparoscopic cholecystectomy performed for a presumed benign gallbladder polyp. This case presents the clinical course of an early-stage, well-differentiated gallbladder NET and provides additional data to the limited body of literature on this rare entity.

## Case presentation

A 39-year-old man presented to the emergency department with right upper quadrant (RUQ) abdominal pain. His medical history included chronic myeloid leukemia, for which he had been receiving dasatinib (70 mg/day) for seven years. Physical examination revealed localized tenderness in the RUQ without rebound tenderness or guarding.

**Figure 1:**
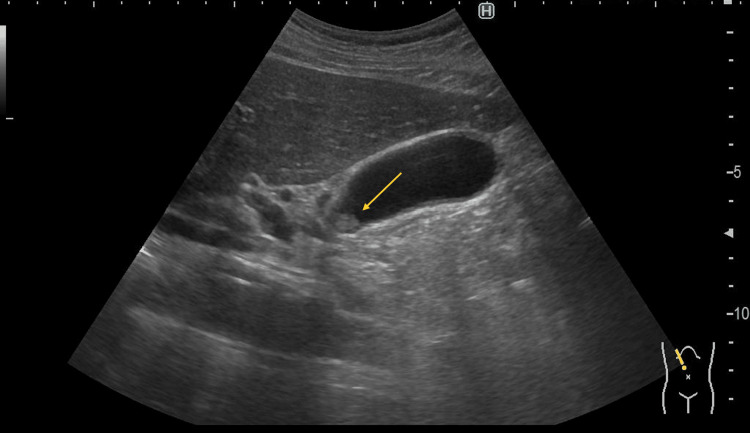
Abdominal US Findings Abdominal US revealed a hypoechoic mass measuring 9 mm in the gallbladder neck (arrow). The surrounding gallbladder wall showed thickening consistent with chronic cholecystitis.

Laboratory findings showed no evidence of systemic inflammation, and liver and biliary enzyme levels were within normal limits. Serum tumor markers, including carcinoembryonic antigen and carbohydrate antigen 19-9, were also within normal ranges. Abdominal ultrasonography (US) revealed a 9 mm polypoid lesion in the neck of the gallbladder, without evidence of a sessile morphology or gallstones (Figure 1).

**Figure 2:**
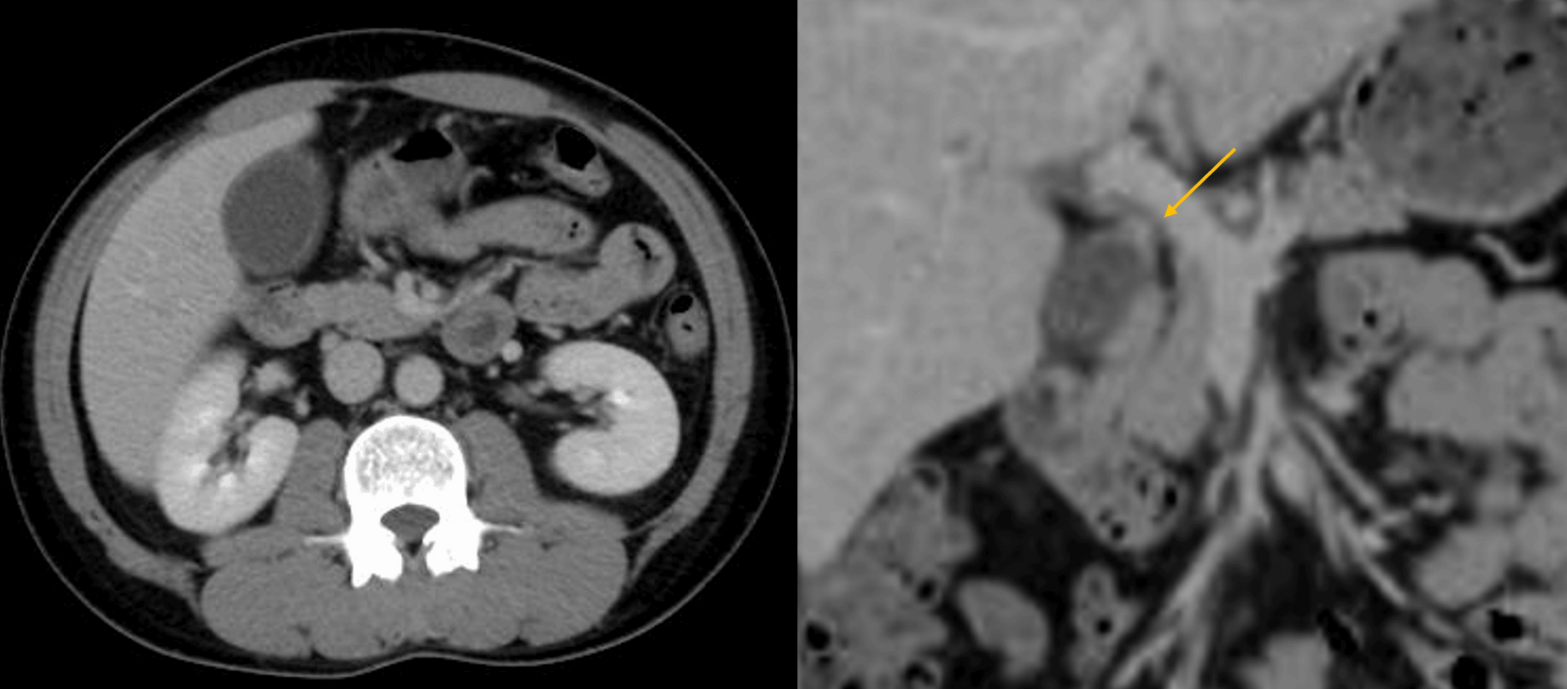
Findings of Abdominal CT Abdominal CT demonstrated gallbladder distension and wall thickening and revealed a mildly enhancing mass at the cystic duct junction (arrow).

Contrast-enhanced computed tomography (CT) demonstrated an 8 mm slightly enhancing lesion in the gallbladder neck, along with gallbladder wall thickening and distension consistent with cholecystitis (Figure 2).

**Figure 3:**
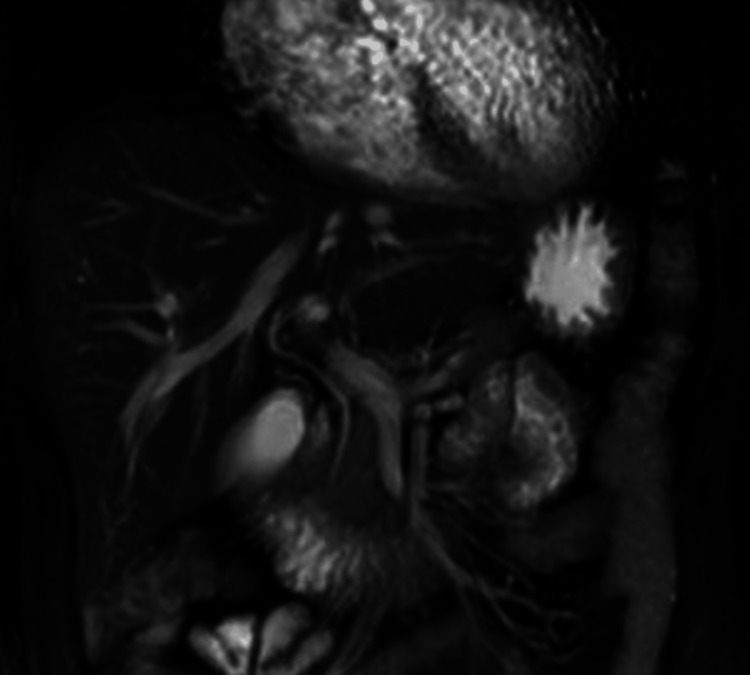
Findings of MRCP Magnetic resonance cholangiopancreatography (MRCP) failed to visualize the lesion due to its small size and showed no abnormalities in the bile or pancreatic ducts.

There was no evidence of hepatic invasion, lymph node enlargement, or distant metastasis. Magnetic resonance cholangiopancreatography (MRCP) failed to visualize the lesion due to its small size and showed no abnormalities in the bile or pancreatic ducts (Figure 3).

Based on these findings, the patient did not initially meet the clear indications for surgery as there was no evidence of systemic inflammation and the polyp measured only 9 mm. However, given its location near the cystic duct junction, intermittent obstruction of the cystic duct by the polyp was suspected, which may have caused biliary colic-like attacks. The patient continued to experience recurrent attacks during follow-up. Therefore, laparoscopic cholecystectomy (LC) was performed five months after the initial visit under a preoperative diagnosis of a benign gallbladder polyp. The postoperative course was uneventful, and the patient was discharged on postoperative day 3.

**Figure 4:**
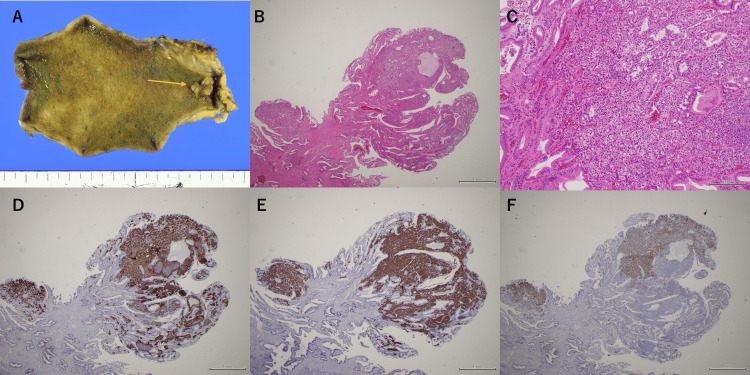
Gross and histopathological findings of the resected gallbladder specimen. (A) Gross examination revealed a semi-pedunculated polyp located in the gallbladder neck (arrow). (B, C) Histopathological examination showed uniform proliferation of tumor cells confined to the mucosa, with occasional glandular structures (H&E staining; original magnification ×40 and ×200). (D) The tumor cells were positive for chromogranin A (×40). (E) The tumor cells were positive for synaptophysin (×40). (F) The tumor cells were partially positive for CD56 (×40).

Gross examination of the resected specimen revealed a semi-pedunculated polyp measuring 10 mm in diameter located in the gallbladder neck. Histopathological examination demonstrated uniform proliferation of tumor cells confined to the mucosa, forming occasional glandular structures, with background chronic cholecystitis. Immunohistochemically, the tumor cells were positive for neuroendocrine markers, including chromogranin A, synaptophysin, and CD56, and the Ki-67 labeling index was <1% (Figure 4).

Based on these findings, the final diagnosis was neuroendocrine tumor (NET) G1 of the gallbladder [4]. Given the low grade of the tumor, no adjuvant therapy was considered necessary. Postoperatively, the patient was followed with periodic abdominal imaging, including US and CT, at regular intervals. No evidence of recurrence or metastasis has been observed during a follow-up period of five years, and the patient remains recurrence-free to date.

## Discussion

NENs, formerly referred to as carcinoid tumors, are now classified according to the WHO grading system based on the Ki-67 labeling index and mitotic count, which correlate well with biological behavior and prognosis. The 2022 WHO classification categorizes these neoplasms into well-differentiated NETs, which are further graded as G1, G2, or G3 based on their proliferative activity, and poorly differentiated NECs (small- or large-cell type). Moreover, mixed tumors containing both neuroendocrine and non-neuroendocrine components, previously termed mixed adeno-neuroendocrine carcinomas (MANECs), are now designated as mixed neuroendocrine-non-neuroendocrine neoplasms (MiNENs) [4,5].

NENs are rare overall, and those originating from the gallbladder (gallbladder NENs) are particularly uncommon, representing only about 0.5% of all NENs [2,3,6]. Approximately 60% of NENs arise in the gastrointestinal tract, followed by the lungs [1,7]. The scarcity of neuroendocrine and enterochromaffin-like cells in the biliary epithelium may explain the rarity of gallbladder NENs. Chronic inflammation has been suggested to induce metaplastic changes leading to the emergence of neuroendocrine cells, which may subsequently give rise to gallbladder NENs [8]. In mixed tumors with adenocarcinoma components, neuroendocrine differentiation may develop secondarily within pre-existing adenocarcinoma lesions [6]. Risk factors such as gallstones or an anomalous pancreaticobiliary ductal junction, both associated with chronic cholecystitis, have also been implicated in the pathogenesis of gallbladder NENs [9]. In the present case, although neither gallstones nor ductal anomalies were observed, histological findings demonstrated chronic cholecystitis, suggesting that persistent inflammation may have played a role in tumorigenesis. Although the patient had a history of chronic myeloid leukemia and long-term dasatinib therapy, there are currently no well-established associations between these factors and gallbladder NENs. In the absence of evidence suggesting a hereditary syndrome or drug-related tumorigenesis, chronic cholecystitis remains the most plausible contributing factor in this case.

Gallbladder NENs are typically asymptomatic or present with nonspecific abdominal pain. The gallbladder neck is the most common site of origin [9]. Many gallbladder NENs exhibit a polypoid or submucosal appearance on imaging. Unlike NENs of the extrahepatic bile duct, gallbladder NENs rarely cause obstructive jaundice unless they are locally advanced. Carcinoid syndrome is extremely rare in gallbladder NENs [6,9,10], as observed in our patient.

Preoperative diagnosis of gallbladder NENs is challenging. While endoscopic or image-guided biopsy, such as endoscopic ultrasonography-guided fine-needle aspiration (EUS-FNA), can confirm intra-abdominal NENs, tissue sampling is often difficult for gallbladder lesions due to anatomical limitations [10-12]. Furthermore, intratumoral heterogeneity may result in discrepancies between biopsy and surgical specimens. For instance, a previous report on pancreatic NENs demonstrated an 83% concordance rate in the Ki-67 index between EUS-FNA and resected samples [13]. In our case, because the lesion was presumed to be a benign gallbladder polyp, preoperative tissue sampling was not performed.

From a clinical standpoint, current management strategies for gallbladder polyps are largely based on size criteria. Although surveillance is generally recommended for polyps smaller than 10 mm in the absence of high-risk features, the present case illustrates that additional factors should be considered. In this patient, recurrent cholecystitis-like symptoms, localized gallbladder wall thickening, and the polyp’s location near the cystic duct junction prompted surgical intervention despite the small size of the lesion. EUS, with or without FNA, may be considered in selected cases with equivocal imaging findings or discordance between polyp size and clinical features. However, given the technical challenges of tissue sampling in gallbladder lesions, cholecystectomy can serve as both a diagnostic and therapeutic approach in symptomatic patients.

Imaging findings of gallbladder NENs are nonspecific; these lesions often appear as well-enhancing masses on contrast-enhanced CT, making them difficult to distinguish from gallbladder carcinoma. Early-stage gallbladder NENs, such as in our patient, are particularly difficult to identify radiologically [10].

Surgical resection remains the mainstay of treatment for resectable gallbladder NENs, with the surgical approach (simple cholecystectomy vs. extended resection with lymphadenectomy or hepatectomy) determined by tumor invasion and differentiation [8,9]. In well-differentiated NETs, curative resection generally yields favorable long-term outcomes [9], whereas poorly differentiated NECs are associated with aggressive behavior and poor prognosis, even after resection. In the present case, LC was performed because the preoperative diagnosis was a benign gallbladder polyp. Postoperative pathological examination revealed a well-differentiated NET G1 confined to the mucosa, with no invasion into the muscular layer, negative surgical margins, and no evidence of lymphovascular or perineural invasion. No lymph node metastasis was identified. Based on these favorable pathological features, simple cholecystectomy was considered adequate a posteriori, and no additional surgical intervention was required. If any NEN or other malignancy is suspected preoperatively, intraoperative frozen-section examination and extended cholecystectomy with lymph node dissection should be considered. However, this case suggests that simple cholecystectomy may be sufficient for selected patients with early-stage, well-differentiated NETs lacking high-risk pathological features.

Due to the rarity of gallbladder NENs, their long-term prognosis remains incompletely understood [14]. Earlier reports likely included a heterogeneous mixture of NETs, NECs, and MiNENs, as older classifications did not clearly distinguish among these entities [15,16]. Consequently, only a limited number of well-differentiated NET G1 cases of the gallbladder, particularly those detected at an early stage, have been described in the literature.

In gastrointestinal NETs, the reported 5-year survival rates range from 88% to 93% for NET G1 and 44% to 82% for NET G2 [17-19], indicating a generally favorable prognosis for low-grade tumors. However, whether these outcomes can be directly extrapolated to gallbladder NENs remains uncertain because of their rarity and biological heterogeneity. To date, reports of mucosa-limited gallbladder NET G1 smaller than 10 mm treated with simple cholecystectomy and accompanied by long-term follow-up are extremely limited. In this context, our case adds valuable information, as the tumor was confined to the mucosa, completely resected by LC, and showed no high-risk pathological features.

These findings suggest that, although long-term surveillance remains warranted even for low-grade gallbladder NETs, early-stage, well-differentiated tumors without adverse pathological features may achieve excellent long-term outcomes after simple cholecystectomy alone.

## Conclusions

Gallbladder NETs are rare neoplasms, and their preoperative diagnosis remains challenging due to nonspecific clinical and radiological findings. Chronic inflammation may play a role in their pathogenesis, even in the absence of gallstones or biliary tract anomalies. In the present case, a well-differentiated NET G1 was successfully treated with LC, with no recurrence observed over a five-year follow-up period. Given the scarcity of such early-stage cases, further accumulation of clinical data is essential to establish optimal management strategies and clarify the long-term biological behavior of this rare entity.
